# Phenolic Profile and Comparison of the Antioxidant, Anti-Ageing, Anti-Inflammatory, and Protective Activities of *Borago officinalis* Extracts on Skin Cells

**DOI:** 10.3390/molecules28020868

**Published:** 2023-01-15

**Authors:** Monika Michalak, Martyna Zagórska-Dziok, Marta Klimek-Szczykutowicz, Agnieszka Szopa

**Affiliations:** 1 Department of Dermatology, Cosmetology and Aesthetic Surgery, Medical College, Jan Kochanowski University, IX Wieków Kielc 19, 35-317 Kielce, Poland; 2 Department of Technology of Cosmetic and Pharmaceutical Products, Medical College, University of Information Technology and Management in Rzeszow, Kielnarowa 386a, 36-020 Tyczyn, Poland; 3 Chair and Department of Pharmaceutical Botany, Faculty of Pharmacy, Medical College, Jagiellonian University, Medyczna 9, 30-688 Kraków, Poland

**Keywords:** *Borago officinalis*, cytotoxicity, polyphenols, antioxidant, anti-inflammatory, anti-collagenase, anti-elastase, skin

## Abstract

In this study, methanol and water–methanol extracts of borage (*Borago officinalis*) herb dried using various methods were analysed for their phenolic profile and biological activity. Twelve compounds, including flavonoids (astragalin, kaempferol 4-glucoside, rutoside, and vitexin) and phenolic acids (caffeic, chlorogenic, 3,4-dihydroxyphenylacetic, ferulic, *p*-hydroxybenzoic, protocatechuic, rosmarinic, and syringic), were determined qualitatively and quantitatively in *B. officinalis* extracts by the HPLC-DAD method. The highest total flavonoid content was confirmed for the methanol extract from the hot-air-dried herb, while the methanol extract from the air-dried herb was most abundant in phenolic acids. The results of in vitro tests on human keratinocytes (HaCaT) and fibroblasts (BJ) showed that the extracts were able to reduce the intracellular level of reactive oxygen species in skin cells. Tests performed to assess inhibition of protein denaturation, lipoxygenase activity, and proteinase activity demonstrated that borage extracts have anti-inflammatory properties. In addition, the methanol extract of the herb dried in a convection oven showed the strongest inhibition of both collagenase and elastase activity, which is indicative of anti-ageing properties. The results show that the borage extracts are a source of valuable bioactive compounds with beneficial properties in the context of skin cell protection.

## 1. Introduction

Over the centuries, medicinal plants have been used for many purposes: in medicine and nutrition, as diet supplements, flavourings, fragrances, and dyes, as well as in cosmetics [1]. An example of a plant with high medicinal and nutritional value and multiple applications is *Borago officinalis* L. (borage, starflower, Boraginaceae). According to literature data, borage is used as a health-improving agent due to its various biological activities. The results of some studies indicate that it can be used as a supportive treatment in respiratory, urinary, and skin disorders, as well as in cardiovascular and inflammatory diseases [1,2]. Borage is an annual blooming herb from June to July native to the Mediterranean region [1]. It is grown in Europe, South America, and Asia [1,3]. *B. officinalis* is a plant covered in rough hairs, with a thick stem that grows to a height of 60–100 cm, alternate and simple leaves that smell of cucumber, and pink, light blue, or less often white flowers [3,4]. The herbal material is the herb (*Boraginis herba*) and seeds (*Boraginis semen*), from which borage oil is obtained [2,3]. Owing to its high content of gamma-linolenic acid (GLA), the oil is valued as a food and pharmaceutical raw material [3]. The herb is recommended for external use because it contains toxic pyrrolizidine alkaloids (lycopsamine, supinidine, amabiline, and intermedine), mainly affecting the liver parenchyma [2]. Phytochemical analyses indicate that borage herb contains carbohydrates, fatty acids, phytosteroids, polyphenols (including vanillic, p-coumaric, p-hydroxybenzoic, gentisic, caffeic, sinapic, rosmarinic, and chlorogenic acids, quercetin, isorhamnetin, and kaempferol), tannins, saponins, mucous compounds, organic acids (ascorbic, malic, citric, acetic, and lactic acid), tocopherols, allantoin, mineral salts, and vitamins, as well as volatile oil [2,3,4,5,6]. Due to its content of active substances, *B. officinalis* herb extract can be used in topical skin products with antioxidant, anti-inflammatory, anti-ageing, UV-protective, soothing, or softening effects [3,5]. An important group of compounds present in borage herb is polyphenols, known for their ability to scavenge free radicals. The antioxidant properties of phenolic compounds, including phenolic acids, flavonoids, and their derivatives, are associated with the presence of hydroxyl groups bound to an aromatic ring [7]. Literature data indicate that differences in the antioxidant capacity of borage may be due in part to the type of extract, as well as qualitative and quantitative variability in the phenolic constituents of the extracts [6]. Plant polyphenols are considered important substances for skin function. Antioxidants have been shown to reduce ROS-induced symptoms of photoageing of the skin. Moreover, polyphenols inhibit the activity of enzymes present in skin-collagenase and elastase, which catalyse hydrolysis of collagen and elastin fibres [7,8]. A review of the literature reveals that borage is used not only as a plant material with antioxidant properties [1,5] but can also be exploited in treatment of chronic inflammatory skin diseases, such as psoriasis vulgaris or atopic dermatitis [2,3]. Borage seed oil has been shown to have potential anti-inflammatory effects, mainly due to the presence of gamma-linolenic acid (GLA) in borage seed oil, as in the seed oil of some other plants [3]. Moreover, borage juice is believed to remove inflammation and support skin regeneration, and ointment prepared from the fresh herb has been found to be effective at treating eczema and healing wounds [2].

To the best of our knowledge, research has focused mainly on borage seed extracts, while studies on different borage herb extracts are limited. Therefore, the aim of the present study was to investigate the potential antioxidant, anti-ageing, and anti-inflammatory properties of *B. officinalis* herb extracts. In addition, experiments were performed to assess the cytotoxicity of the tested extracts. In this work, two human cell lines, keratinocytes (HaCaT) and fibroblasts (BJ), were used. Moreover, the corresponding phenolic compounds’ qualitative and quantitative profiles of studied herb extracts using the HPLC-DAD method were assessed. The innovative nature of the work lies in comprehensive analyses of borage herb extracts using epidermal and dermal cells as potential material for topical application. To the best of our knowledge, no research has been conducted to assess the antioxidant, anti-ageing, and anti-inflammatory properties of borage extracts on HaCaT and BJ cell lines. The present study makes a significant contribution to the current search for new natural materials, or new applications of those already known, that could replace synthetic materials. This is particularly important in the case of treatment of chronic inflammatory skin diseases, in which a key role is played by long-term application of substances that are safe for the skin and exert multifaceted effects. Another important aspect of the research is assessment of the biological properties of borage extracts in relation to their content of polyphenolic compounds.

## 2. Results and Discussion

### 2.1. Phenolic Profiling

**Figure 1:**
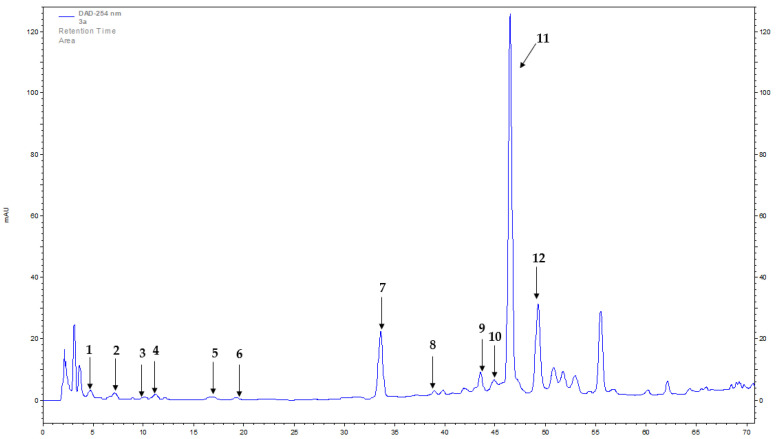
HPLC-DAD (λ = 254 nm, UV spectra range 200–400 nm) chromatogram of hot-air-dried herb methanol extract of *B. officinalis*; (1) protocatechuic acid, (2) 3,4-dihydroxyphenylacetic acid, (3) *p*-hydroxybenzoic acid, (4) chlorogenic acid, (5) caffeic acid, (6) syringic acid, (7) ferulic acid, (8) vitexin, (9) kaempferol 4-glucoside, (10) rutoside, (11) rosmarinic acid, (12) astragalin.

The presence of four flavonoids (astragalin, kaempferol 4-glucoside, rutoside, and vitexin) and eight phenolic acids (caffeic, chlorogenic, 3,4-dihydroxyphenylacetic, ferulic, *p*-hydroxybenzoic, protocatechuic, rosmarinic, and syringic) was confirmed in extracts from *B. officinalis* herb using HPLC-DAD (high-performance liquid chromatography with diode-array detection) method (Table 1 and Table 2, Figure 1). The content of flavonoids and phenolic acids varied depending on the plant material drying method used and the type of extract.

Among flavonoids, the dominant compounds in all extracts of *B. officinalis* were astragalin (max. 248.60 mg/100 g dried weight (DW), methanol extract of hot-air-dried herb), kaempferol 4-glucoside (max. 141.08 mg/100 g DW, methanol extract of hot-air-dried herb), and rutoside (max. 95.71 mg/100 g DW, methanol extract of hot-air-dried herb). The total content of flavonoids, depending on the drying method and extract, ranged from 131.86 to 528.23 mg/100 g DW. The highest flavonoid content was obtained for the methanol extract of *B. officinalis* herb dried in a convection oven. The lowest content was obtained for water–methanol extracts of hot-air-dried herb. The content of all flavonoids was higher in the methanol than water–methanol *B. officinalis* extracts (Table 1).

Among phenolic acids, the dominant compounds in all *B. officinalis* extracts were rosmarinic acid (max. 1783.55 mg/100 g DW, methanol extract of hot-air-dried herb), 3,4-dihydroxyphenylacetic acid (max. 260.17 mg/100 g DW, water–methanol extract of air-dried herb), and ferulic acid (max. 205.30 mg/100 g DW, methanol extract of hot-air-dried herb). The total content of phenolic acids, depending on the drying method and extract, ranged from 489.04 to 2281.19 mg/100 g DW. The highest total content was obtained for the methanol extract of *B. officinalis* herb dried in a convection oven. The lowest content was obtained for water–methanol extracts of hot-air-dried herb (Table 2).

Methanol extracts of *B. officinalis* dried in a convection oven proved to be the most abundant in phenolic compounds from flavonoids and phenolic acids (Table 1 and Table 2).

There are few studies on composition of flavonoids and phenolic acids in *B. officinalis.* In 2017, methanol extracts of freeze-dried flowers were analysed for their content of flavonoids and phenolic acids. Analyses with the RP-HPLC method revealed the presence of compounds such as caffeic, gallic, and salicylic acid, daidzein, myricetin, pyrogallol, and rutoside in the extracts [1]. Similarly, our study of herb extracts also confirmed the presence of caffeic acid and rutoside. Other phenolic acids detected in the herb extracts were chlorogenic, 3,4-dihydroxyphenylacetic, ferulic, *p*-hydroxybenzoic, protocatechuic, rosmarinic, and syringic (Table 2). Among flavonoids, in addition to rutoside, our study demonstrated the presence of astragalin, kaempferol 4-glucoside, and vitexin (Table 1). In 2019, Zemmouri et al. [6] performed a study on aqueous and 80% ethanol extracts of air-dried leaves of *B. officinalis*. Identified in the aqueous extracts with LC/MS assays were phenolic compounds such as caffeic acid, *m*-geranyl-*p*-hydroxybenzoic acid, *p*-hydroxyphenyl lactic acid, *p*-hydroxybenzoic acid glucoside, lithospermic acid B, sinapinic acid hexoside, and syringaldehyde. Among flavonoids, isovitexin, isoquercetin, naringenin *O*-hexosides, luteolin 7-*O*-glucoside, quercetin, and vitexin were identified. Compounds identified in the ethanol extracts included caffeoyl shikimate acid, dihydroferulic acid, 3,4-dimethoxycinnamic acid, *m*-geranyl-*p*-hydroxybenzoic acid, and *p*-hydroxybenzoic acid glucoside, and, among flavonoids, kaempferol 3,7,4′-trimethyl ether, luteolin 7,3′,4′-trimethyl ether, and naringenin *O*-hexosides. The results of that study differ from ours, possibly because the extracts were prepared via a different method. Our study also confirmed the presence of vitexin in the herb extracts, but we additionally identified other flavonoids (astragalin, kaempferol 4-glucoside, rutoside), listed in Table 1, which were not reported by Zemmouri et al. [6]. Comparison of our findings with those of Zemmouri et al. also revealed differences in the presence of phenolic acids in the herb and leaf extracts. In the herb extracts, in addition to caffeic and *p*-hydroxybenzoic acids, we confirmed the presence of six other phenolic acids: chlorogenic, 3,4-dihydroxyphenylacetic, ferulic, protocatechuic, rosmarinic, and syringic (Table 2). In 2020, Kareem et al. [4] studied aerial plants harvested from natural sites in Iraq. The plant material was dried in the dark for two weeks and milled in a mechanical grinder. Cold extraction was performed with 85% methanol for three days. To separate compounds according to polarity, fractionation with petroleum ether, chloroform, ethyl acetate, and n-butanol was carried out. Phenolic compounds were identified and isolated by TLC (thin-layer chromatography), PLC (preparative liquid chromatography), and LC/MS (liquid chromatography–mass spectrometry) methods. The presence of caffeic, rosmarinic, and sinapinic acids was detected in the study [4]. In our study, the phenolic acids identified also included caffeic and rosmarinic acids. In addition, our analyses confirmed the presence of six other phenolic acids (chlorogenic, 3,4-dihydroxyphenylacetic, ferulic, *p*-hydroxybenzoic, protocatechuic, and syringic) (Table 2).

### 2.2. Effect of B. officinalis Extracts on Skin Cell Viability

**Figure 2:**
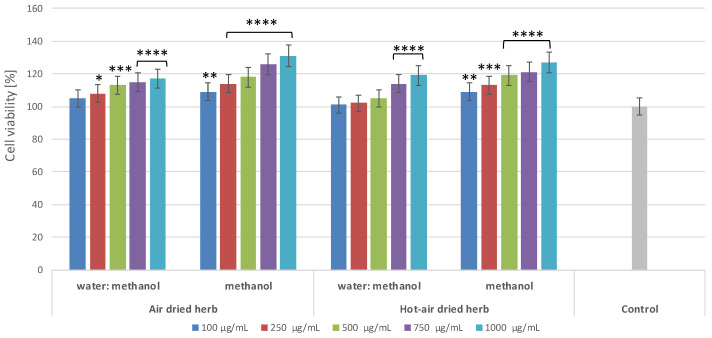
Reduction of resazurin after exposure to *B. officinalis* extracts in cultured HaCaT cells. Data are the means ± SD of three independent experiments, each consisting of three replicates per treatment group. * *p* = 0.033, ** *p* < 0.01, *** *p* < 0.001, **** *p* < 0.0001 versus the control (100%).

**Figure 3:**
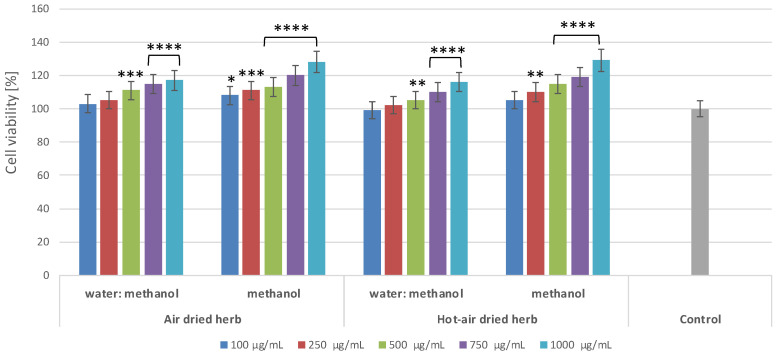
Reduction of resazurin after exposure to *B. officinalis* extracts in cultured fibroblasts. Data are the means ± SD of three independent experiments, each consisting of three replicates per treatment group. * *p* = 0.065, ** *p* < 0.01, *** *p* < 0.001, **** *p* < 0.0001 versus the control (100%).

To assess the cytotoxic properties of the extracts, a commonly used test based on measurement of resazurin reduction was used. The degree of reduction of this dye reflects the activity of mitochondrial enzymes, which indicates the degree of viability of the cells exposed to the test samples. The results show that the influence of the extracts on the viability of keratinocytes (Figure 2) and fibroblasts (Figure 3) depends on both the type of extract and the concentration used.

The extracts analysed in this paper have not previously been tested for their toxicity to skin cells, yet it is important that substances dedicated for external use should be non-toxic, especially for skin cells, such as keratinocytes and fibroblasts. None of the extracts were shown to exert a cytotoxic effect at any of the concentrations used. Moreover, the extracts positively affected the viability and metabolic activity of both types of skin cells in vitro, increasing it by almost 30% at a concentration of 1000 µg/mL. There were no significant differences between the extracts obtained from material dried using different methods; however, the results indicate that methanol extracts have a more favourable effect on cell viability than water–methanol extracts (Figure 2 and Figure 3). Water–methanol extracts were shown to be less rich in flavonoids than methanol extracts. The results indicate that all the borage extracts tested in the study may have a positive effect on skin cell viability, which is undoubtedly linked to their wide range of biologically active compounds, whose presence was confirmed in the chromatographic analysis. Previous research has shown that flavonoids and phenolic acids present in borage extracts exhibit the ability to increase cell proliferation. For example, rutin exerts cytoprotective effects on cells exposed to various types of radiation, substantially increasing their viability [9]. Rosmarinic acid, predominant in methanolic extracts of borage herb, can exhibit cytoprotective activity, increasing the viability of HaCaT cells owing to its ability to be absorbed within the UVB range [10]. There are also a few studies on the effects of the main active ingredients of borage extracts on cancer cells. For example, polyphenolic compounds, such as astragalin, kaempferol, caffeic, ferulic, and syringic acid, have anti-tumour properties and are able to prevent proliferation of cancerous cells [11,12,13]. Astragalin has been shown to inhibit growth of liver cancer [14], as well as to inhibit proliferation of lung cancer [15] and melanoma cells [11]. Ferulic acid is believed to present low toxicity and many biological properties, including anticancer properties, particularly with respect to lung, breast, colon, and skin cancer [13]. In the case of borage extracts, methanolic, ethanolic, and water flower extracts have been shown to exhibit low anticancer properties against human hepatic, prostate, and colon cancer cells [1].

### 2.3. Intracellular ROS Levels in Skin Cells

**Figure 4:**
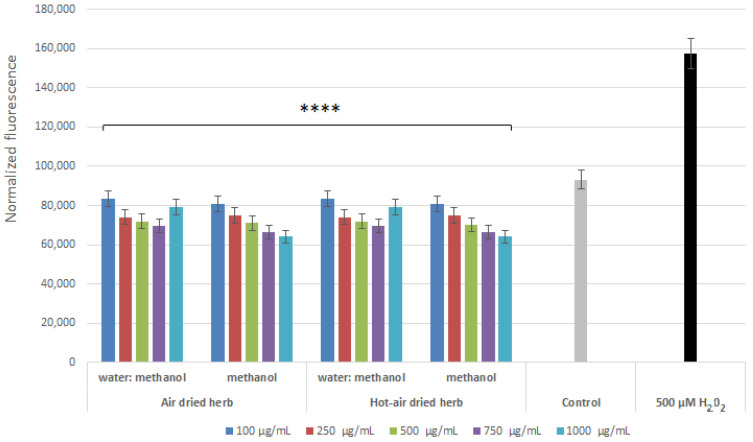
Effect of *B. officinalis* extracts on DCF fluorescence in HaCaT cells. Data are the means ± SD of three independent experiments. **** *p* < 0.0001 versus the control.

**Figure 5:**
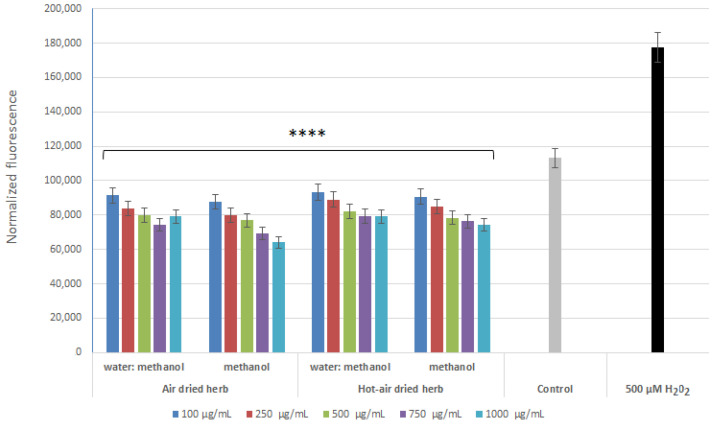
Effect of *B. officinalis* extracts on DCF fluorescence in BJ cells. Data are the means ± SD of three independent experiments. **** *p* < 0.0001 versus the control.

Analyses aimed at assessing the level of reactive oxygen species (ROS) in cells treated with hydrogen peroxide showed that the extracts were able to reduce the level of ROS caused by the action of H_2_O_2_. This effect was observed in both keratinocytes (Figure 4) and fibroblasts (Figure 5). All tested types of extracts were able to reduce the level of free radicals in the cells to even below the level in the control (cells not treated with the extract or H_2_O_2_). As the concentration of all four extracts increased to 750 µg/mL, the antioxidant properties of the test samples increased. In the case of fibroblasts, the strongest antioxidant properties were observed for the methanol extract obtained from air-dried herb, while, in the case of keratinocytes, no significant differences were observed between the two methanol extracts. Analyses carried out with the H_2_DCFDA fluorescent probe showed slightly better antioxidant properties for the methanol extracts, most likely due to the higher content of biologically active compounds confirmed in chromatographic analysis.

Numerous studies suggest a correlation between content of phenolic compounds and antioxidant properties of plant extracts. The antioxidant and antiradical properties of polyphenols result from elimination of ROS through direct reactions, scavenging, or reduction of free radicals to compounds with much lower reactivity [7]. The antioxidant activity of *B. officinalis* was assessed using various in vitro assays, including determination of DPPH scavenging activity [1,5,6,16,17], ferric reducing antioxidant power (FRAP) [1,16], oxygen radical antioxidant capacity (ORAC) [16], and scavenging of superoxide radical (NBT test) [6]. These assays of in vitro antioxidant activity were used to analyse methanolic extracts of borage leaves [5], water and ethanolic extracts of the leaves [6], ethanolic extracts of defatted seeds [18], methanolic, ethanolic, and hot water extracts of flowers [1], and also fresh flowers [16]. In addition, borage flowers were tested in vitro on mouse neuroblastoma cells (N2a cells), including assays of intracellular ROS production and endogenous antioxidant enzymes (catalase and superoxide dismutase) [16]. Seo et al. investigated the inhibitory effect of borage ethanolic extract against ROS generation in UVB-irradiated normal human dermal fibroblasts (NHDFs) in vitro [17]. In the present study, the antioxidant activity of methanol and water–methanol extracts of borage herb dried using various methods was assessed, and, for the first time, intracellular ROS levels were measured on two human cell lines: keratinocytes (HaCaT) and fibroblasts (BJ).

In the context of skin protection against ROS, plant materials containing bioactive compounds, including flavonoids and phenolic acids with antioxidant properties, are important components of products intended for topical use as free radicals have adverse effects on the epidermis as well as the dermis. ROS directly damage the DNA and lipids of epidermal keratinocytes. They attack the ceramides and destroy sebum components that contribute to formation of products that irritate the skin and cause inflammatory and allergic reactions. In human keratinocytes, oxidative stress caused by ROS can lead to activation of mitogen-activated protein kinase (MAPK) pathways, which affects regulation of various cellular activities (e.g., proliferation, differentiation, inflammatory responses, and apoptosis). At the level of the dermis, ROS can induce expression of proteinases responsible for remodelling the extracellular matrix (ECM), such as serine proteases and matrix metalloproteinases (MMPs), mainly MMP-1 and MMP-3 [7]. Collagenase 1 (MMP-1) has been shown to be responsible for degradation of collagen, whereas stromelysin-1 (MMP-3), which may be induced during wound repair or inflammation, has been implicated in many inflammatory diseases [7,19].

### 2.4. Anti-Inflammatory Properties of B. officinalis

#### 2.4.1. Inhibition of Protein Denaturation

**Figure 6:**
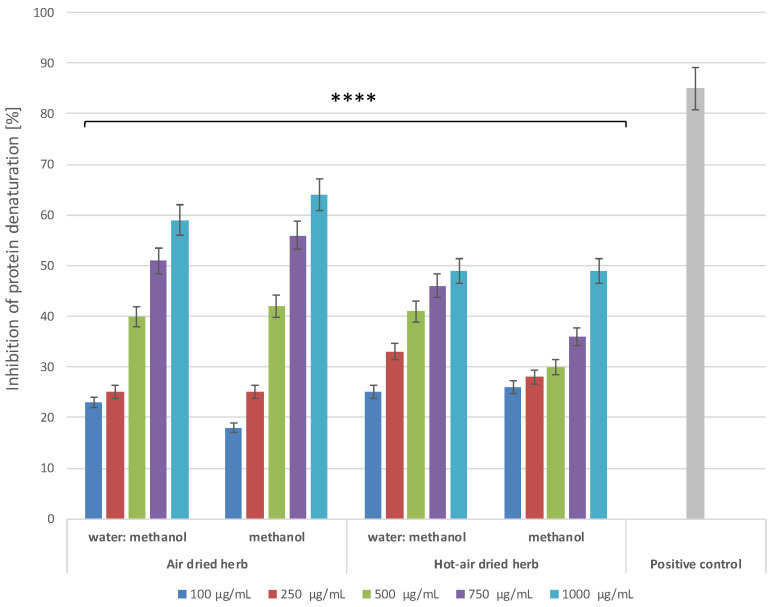
Effect of *B. officinalis* extracts on inhibition of BSA protein denaturation. Data are the means of three independent experiments. **** *p* < 0.0001 versus the control.

An in vitro protein (albumin, BSA) denaturation bioassay was used to evaluate the anti-inflammatory properties of *B. officinalis* herb extracts. Inhibition of protein denaturation, and, thus, the possibility of stabilizing cell membranes, have not yet been fully investigated. However, it has been suggested that this effect may inhibit release of lysosomal components of neutrophils responsible for development of inflammation and tissue damage at the site of inflammation [20,21]. The results showed concentration-dependent and type-dependent inhibition of protein (BSA) denaturation in the concentration range of 100–1000 μg/mL (Figure 6). Non-steroidal anti-inflammatory drug diclofenac, at a concentration of 500 µg/mL, was used as a control inhibitor, which also inhibited BSA denaturation. Comparison of the potential to inhibit protein denaturation between the extracts indicates that those obtained from air-dried herb have stronger anti-inflammatory properties. Inhibition of BSA denaturation reaches over 63% in the case of the methanol extract of air-dried herb and over 58% for the water–methanol extract, while, in the case of extracts obtained from hot-air-dried herb, inhibition does not exceed 50%. Comparison of the results with the level of inhibition of protein denaturation by diclofenac suggests that the extracts are a promising natural material for treatment of inflammation.

#### 2.4.2. Lipoxygenase Inhibitory Activity

**Figure 7:**
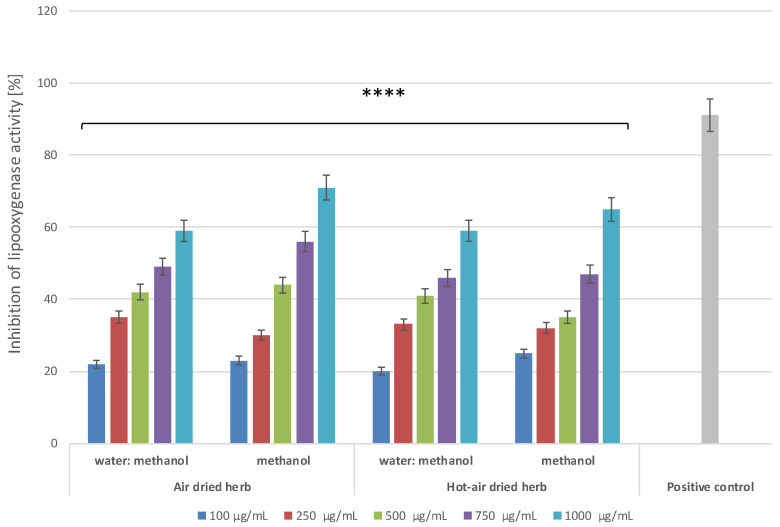
Lipoxygenase inhibitory activity of *B. officinalis* herb extracts. Data are the means of three independent experiments. **** *p* < 0.0001 versus the control.

Lipoxygenase (LOX) is an enzyme that plays an important role in conversion of arachidonic acid to leukotriene, which is closely involved in inflammation. Hence, compounds that can inhibit LOX activity can reduce the amount of leukotriene formed and thus reduce inflammation. Lowering the leukotriene level may reduce the number of induced and recruited populations of pro-inflammatory cells, which may significantly mitigate the negative effects of inflammation [22]. Therefore, the potential anti-inflammatory properties of the extracts were also tested by evaluating their capacity to inhibit the activity of this enzyme. As with the protein denaturation inhibition assay, dose-dependent and extract-type-dependent inhibition of LOX activity was demonstrated (Figure 7). The greatest inhibition was observed for methanol extracts at a concentration of 1000 µg/mL, with more than 70% inhibition for the air-dried herb extract and more than 60% for the hot-air-dried herb extract. Comparison of the activity of the extracts with that of diclofenac, for which the inhibition level of LOX activity was slightly over 90%, suggests that the tested extracts are promising inhibitors of this enzyme.

#### 2.4.3. Proteinase Inhibitory Activity

**Figure 8:**
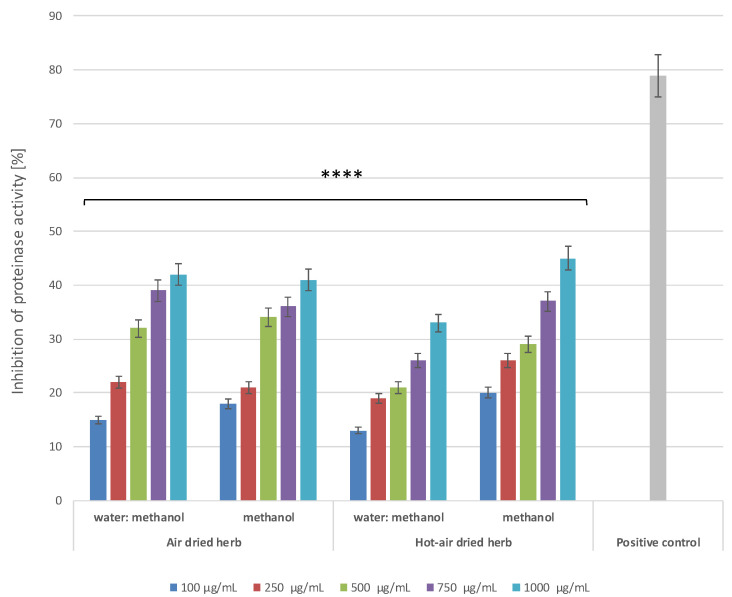
Proteinase inhibitory activity of *B. officinalis* herb extracts. Data are the means of three independent experiments. **** *p* < 0.0001 versus the control.

Leukocyte proteinases play an extremely important role in development of tissue damage associated with ongoing inflammatory processes. Hence, compounds showing the ability to inhibit proteinases can play a significant role in inhibiting inflammation [21]. Literature data indicate that many biologically active compounds present in plants can inhibit the activity of these enzymes [23,24], so this work also assessed the possibility of proteinase inhibition. The results indicated that all four extracts can inhibit the activity of this enzyme in a manner dependent on both the concentration and type of extractant (Figure 8). For both types of extracts obtained from air-dried herb and for the methanol extract from hot-air-dried herb, inhibition of more than 40% was obtained at the highest concentration tested.

A review of the literature shows that previous research has tested the anti-inflammatory properties of *B. officinalis* seed oil [25], hydroalcoholic extract of the leaves [26], methanolic, ethanolic, and water extracts of the flowers [1], and ethanolic extracts of the herb [17]. Asaad et al., in a study using mature Wistar rats and mature albino mice, confirmed the anti-inflammatory properties of *B. officinalis* seed oil [15]. Barati et al. investigated the effect of borage on hippocampal interleukin 1 beta (IL-1β) in beta-amyloid-peptide-induced inflammation in Wistar rats [26]. Karimi et al. reported that the flower methanolic, ethanolic, and water borage extracts showed weak anti-inflammatory activity in murine RAW 264.7 macrophage cells [1]. Seo et al. demonstrated that borage can inhibit interleukin 6 (IL-6) production and promote *transforming growth factor beta* (TGF-β1) production in UVB-irradiated normal human dermal fibroblasts (NHDFs) [17]. No previous paper has studied the anti-inflammatory effect of methanol and water–methanol extract of borage herb using human *skin keratinocytes* (*HaCaT*) and fibroblasts (BJ). The combined results of the three in vitro tests (inhibition of protein denaturation and lipoxygenase and proteinase inhibitory activity) suggest that *B. officinalis* herb extracts can, to some extent, inhibit inflammatory reactions. These findings support the view that borage can be used in chronic inflammatory skin diseases [2,3]. The analyses presented in this study, aimed at assessing the anti-inflammatory properties of the extracts, showed a significant correlation between these properties and the content of biologically active compounds, such as flavonoids and phenolic acids. The differences observed between types of extracts indicate that extracts with a higher content of flavonoids have stronger anti-inflammatory properties, which demonstrates their important role in fighting inflammation. The anti-inflammatory properties of this group of polyphenolic compounds may be linked to activation of antioxidant pathways, inhibition of enzyme secretion (such as lysozymes and β-glucuronidase), and inhibition of arachidonic acid secretion, resulting in inhibition of inflammatory processes [27,28,29]. Flavonoids can also modulate expression and activation of a wide variety of cytokines, such asIL-1β, TNF-α, IL-6, and IL-8. These compounds have also shown the ability to regulate expression of various pro-inflammatory genes and to inhibit activity of pro-inflammatory enzymes, such as inducible nitric oxide synthase (iNOS), cyclooxygenase 2 (COX-2), and lipoxygenase (LOX) [27,30].

Literature data indicate that some of the phenolic acids present in the analysed borage extracts, such as rosmarinic, syringic, chlorogenic, caffeic, and ferulic acid, can modulate pro-inflammatory gene expression and cytokine production, thus impacting immune cell populations [31]. For instance, rosmarinic acid is able to regulate the immune system by inhibiting inflammation [32], prevents UVB-induced inflammation [33], and attenuates atopic dermatitis, an inflammation-related skin disorder [34]. Syringic acid can suppress UVB-induced COX-2 expression, as well as PGE_2_ production, by suppressing AP-1 transactivation [35].

Due to their content of bioactive compounds and anti-inflammatory effects, *B. officinalis* herb extracts are expected to be applicable in dermocosmetic products as a valuable raw material for improving human skin disorders. This natural plant material can also help in reducing the side effects of non-steroidal anti-inflammatory drugs [25].

### 2.5. Anti-Ageing Properties of B. officinalis

In recent decades, the number of studies on skin ageing has been continually increasing. Since the discovery of collagenase-1, the first matrix metalloproteinase, by Gross and Lapière more than half a century ago, studies on MMPs have provided a great deal of information on these zinc-dependent endopeptidases [19,36]. The main biological function of MMPs is degradation of ECM proteins and glycoproteins, as well as membrane receptors, cytokines, and growth factors. MMPs are involved in many biological processes, including cell proliferation, tissue repair and remodelling, wound healing, and apoptosis [36]. Upregulation of MMPs leads to development of various pathologies, including weakening of the matrix [19,36]. Further research on skin health and ageing should explore MMP function and its impact on physiological and pathological processes and attempt to find new active compounds functioning as MMP inhibitors.

**Figure 9:**
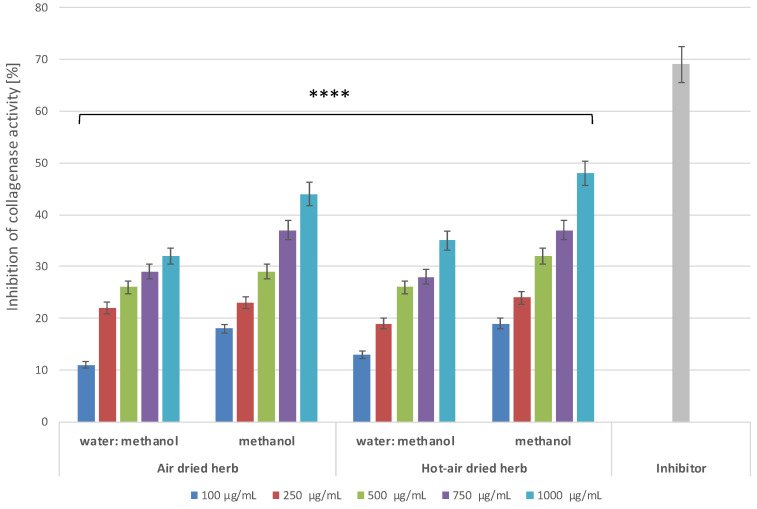
Collagenase inhibitory activity of *B. officinalis* extracts. Data are the means of three independent experiments, each consisting of two replicates per treatment group. **** *p* < 0.0001 versus the control.

**Figure 10:**
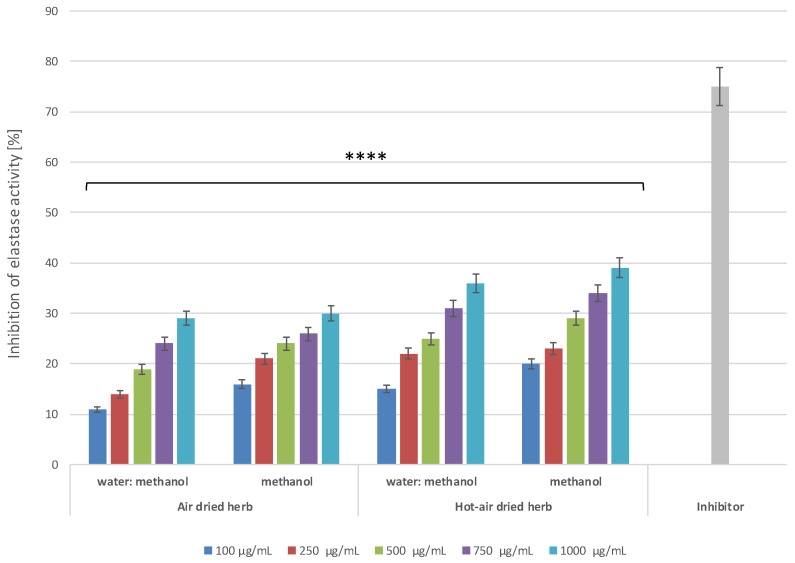
Elastase inhibitory activity of *B. officinalis* extracts. Data are the means of three independent experiments, each consisting of two replicates per treatment group. **** *p* < 0.0001 versus the control.

In the present study, to evaluate the potential anti-ageing properties of *B. officinalis* extracts, experiments were carried out to assess their ability to inhibit activity of collagenase and elastase, proteolytic enzymes responsible for degradation of collagen and elastin fibres—the basic protein components of the skin [37]. Collagen is responsible for the tensile strength of the skin, while elastin fibres ensure its elasticity. Degradation of these proteins causes visible changes in the condition of the skin, manifested mainly by the appearance of wrinkles and changes in its thickness [38,39]. In addition, excessive amounts of ROS also increase the activity of MMPs, such as collagenase and elastase, thus accelerating skin ageing [40]. Suppression of skin-related enzymes degrading structural components of the extracellular matrix (ECM), including collagen and elastin fibres, is believed to be a key strategy in ensuring skin integrity. Hence, finding natural resources that could act as inhibitors of MMPs is extremely important. The results indicate that the tested extracts show concentration-dependent inhibition of the activity of both collagenase (Figure 9) and elastase (Figure 10).

In the case of collagenase, greater inhibition is shown by methanol extracts, for which inhibition at the highest concentration used was over 44% for the air-dried herb extract and 48% for the hot-air-dried herb extract. The analyses assessing the possibility of inhibiting elastase activity showed higher inhibition by extracts from hot-air-dried herb compared to herb dried in the air. In the case of methanol extracts of *B. officinalis* dried in a convection oven, nearly 40% inhibition of elastase activity was observed, while, for extracts of air-dried herb, this inhibition did not exceed 30%.

Comparison of the inhibition of the analysed MMPs by the tested extracts with the values obtained for commonly known inhibitors of these enzymes suggests that these extracts may prove to be valuable tools in the fight against skin ageing. Although enzyme inhibition by the extracts is lower than that of the control inhibitors, it is sufficient to significantly slow down degradation of both collagen and elastin fibres, thus helping to maintain good skin condition.

## 3. Materials and Methods

### 3.1. Plant Material

Experiments were carried out on the aerial parts (herb) of *B. officinalis*. The herb was harvested at the beginning of blooming in 2021 from the Botanical Garden in Kielce, Poland (51°7′ N, 23°28′ E). A voucher specimen of *B. officinalis* was deposited at the Herbarium KPC, Jan Kochanowski University, Kielce, Poland. The collected parts of the plants were immediately subjected to drying by one of two methods: natural air-drying, in a shaded, well-ventilated room at 20–22 °C), and in a laboratory convection oven (Binder FD 53, Tuttlingen, Germany) in a stream of air at 40 °C.

### 3.2. Extract Preparation

Air-dried and hot-air-dried herbs were used to prepare methanol and water–methanol (70:30, *v*/*v*) extracts. For each extract, 2 g of biomass was weighed out and pulverized in an electric grinder (MF 10 basic, IKA-werke, Staufen, Germany). The material was subjected to extraction with 60 mL methanol (STANLAB, Lublin, Poland) for methanol extracts and 42 mL redistilled water with 18 mL methanol (STANLAB, Lublin, Poland) for water–methanol extracts (70:30, *v*/*v*). Extraction was carried out twice under sonication for 20 min in an ultrasonic bath (Polsonic 3, Warsaw, Poland). Then, the extracts were filtered into crystallizers using Whatman paper.

### 3.3. Determination of Bioactive Compounds with HPLC-DAD

Analyses of flavonoids and phenolic acids were carried out using the HPLC-DAD method as described previously [41,42]. The obtained extracts (prepared as described in Section 3.2) were filtered through sterilizing syringe filters (0.22 μm, Millex^®^GP, Merck Millipore, Burlington, MA, USA prior to HPLC analysis. An HPLC-DAD system (Merck-Hitachi, Merck KGaA, Darmstadt, Germany) and a Purospher RP-18e analytical column (4 × 250 mm, 5 µm; Merck, Darmstadt, DE-HE, Germany) were used for the analysis. Elution was completed with a mobile phase A methanol:0.5% acetic acid, 1:4, *v*/*v*) and a mobile phase B (methanol). The gradient program was set as follows: 0–20 min, 0% B; 20–35 min, 0–20% B; 35–45 min, 20–30% B; 45–55 min, 30–40% B; 55–60 min, 40–50% B; 60–65 min, 50–75% B; and 65–70 min, 75–100% B. The injection volume was 10 μL and the compounds of interest were detected at 254 nm. Identification and quantification were performed based on comparison with standard retention times and calibration curves method. The following flavonoid 20 standards were applied: apigenin, astragalin, cymaroside, hyperoside, isoquercetin, kaempferol, kaempferol 4-glucoside, kaempferol 7-rhamnoside, luteolin, myricetin, naringin, populin, quercetin, quercimetrin, quercitrin, rhamnetin, robinin, routine, trifolin, vitexin. The 27 standards of phenolic acids were searching caffeic, caftaric, chlorogenic, m-coumaric, o-coumaric, p-coumaric, cryptochlorogenic, 3,4-dihydroxyphenylacetic, ellagic, ferulic, gallic, gentisic, hydrocaffeic, p-hydroxybenzoic, isochlorogenic, isoferulic, neochlorogenic, phenylacetic, 3-phenylacetic, protocatechuic, rosmarinic, salicylic, sinapic, syringic, and vanillic acids, as well as the precursors: benzoic and cinnamic acids. All compounds purchased from Sigma-Aldrich, Saint Louis, MO, USA.

### 3.4. Cell Culture

Analyses of cytotoxicity of extracts obtained from the *B. officinalis* herb were carried out on two lines of skin cells. Normal human HaCaT keratinocytes (CLS Cell Lines Service GmbH, Eppelheim, Germany) and BJ fibroblasts (American Type Culture Collection, Manassas, VA 20108, MA, USA) were used in the analyses. Cells were grown in Dulbecco’s Modified Eagle Medium (DMEM, Biological Industries, Cromwell, CO, USA), which was supplemented with sodium pyruvate, L-glutamine, and a high glucose content (4.5 g/L). The culture medium was additionally supplemented with 10% foetal bovine serum (Merck Life Science, Darmstadt, Germany) and 1% antibiotics (100 U/mL penicillin and 1000 µg/mL streptomycin) (Gibco, Waltham, MA, USA) to prevent contamination with microorganisms. Both cell types were grown in culture flasks in an incubator at 37 °C in a humidified atmosphere of 95% air and 5% carbon dioxide. Cells were passaged when about 70–80% confluency was achieved.

### 3.5. Assessment of Cytotoxicity—Alamar Blue Assay

A test using a resazurin-based solution (Sigma, R7017, Life Technologies, Bleiswijk, The Netherlands) was used to evaluate the in vitro cytotoxicity of the tested extracts on skin cells. For this purpose, the methodology described earlier by Wójciak et al. [43] was used. In a first step, keratinocytes and fibroblasts were seeded separately in clear 96-well sterile flat-bottom plates (VWR, Radnor, PE, USA) at a density of 1 × 10^4^ cells/well. The cytotoxicity of the analysed extracts was tested at concentrations of 100, 250, 500, 750, and 1000 µg/mL. For this purpose, HaCaT and BJ cells were incubated for 24 h with the tested extracts. After this time, the extract solutions dissolved in the culture medium were aspirated and a 60 µM solution of resazurin was added to each well on the microplate. The prepared plates were incubated at 37 °C for 2 h. After this time, the fluorescence of the solutions in each well was measured with an excitation wavelength of 530 nm and an emission wavelength of 590 nm using a microplate reader (FilterMax F5, Molecular Devices, Silicon Valley, CA, USA, Multi-Mode Analysis Software 3.4.0.25). The control sample was cells (HaCaT and BJ separately) grown in DMEM medium without addition of *B. officinalis* extracts, for which 100% viability was assumed. Three independent experiments were carried out, for which each concentration of the extract was tested in triplicate.

### 3.6. Detection of Intracellular Levels of Reactive Oxygen Species (ROS)

In order to determine the ability of the analysed extracts to inhibit intracellular production of reactive oxygen species in skin cells, tests were performed on human keratinocytes (HaCaT) and fibroblasts (BJ) in vitro based on the methodology previously described by Nizioł-Łukaszewska et al. [44]. For this purpose, fluorogenic cell-permeant indicator H_2_DCFDA was used. As an initial step, cells were seeded in 96-well flat bottom plates at a density of 1 × 10^4^ cells/well and cultured in an incubator for 24 h to attach to the bottom of the wells. The DMEM medium was then removed and replaced with a 10 µM H_2_DCFDA solution (Sigma Aldrich, Sant Louis, MO, USA) dissolved in serum-free DMEM medium. HaCaT and BJ cells prepared in this way were incubated with H_2_DCFDA for 45 min and then treated simultaneously with 500 µM H_2_O_2_ and all four types of extracts in the concentration range of 100–1000 µg/mL. Cells treated with 500 µM hydrogen peroxide (H_2_O_2_) solution were the positive control, while cells untreated with the test extracts were the control. After 60 min, the fluorescence of 2′, 7′-dichlorofluorescein (DCF) was measured in the individual wells containing the cells and treated with the test samples. Measurements were made at excitation wavelength λ = 485 nm and emission λ = 530 nm using a microplate reader (FilterMax F5, Thermo Fisher Scientific, Waltham, MA, USA). As part of the analyses, three independent experiments were performed in which each sample was tested in four replications.

### 3.7. Anti-Inflammatory Activity

#### 3.7.1. Assessment of Inhibition of Protein Denaturation

The anti-inflammatory properties of the analysed *B. officinalis* extracts were assessed based on the methodology previously described by Sarvesvaran et al. [45]. The test used is based on assessment of the possibility of inhibiting denaturation of bovine serum albumin (BSA) by the tested extracts in the concentration range of 100–1000 µg/mL. For this, 1000 µL of individual concentrations of the analysed extracts were mixed with 450 µL of 5% aqueous BSA solution and 1400 µL of phosphate buffered saline (PBS, pH 6.4). The mixtures prepared in this way were incubated in an incubator at 37 °C for 15 min. In the next step, the samples were heated at 70 °C for 5 min and then cooled in an ice bath to 25 °C. Finally, the absorbance of the tested samples was measured at λ = 660 nm using a DR600 UV–vis cuvette spectrophotometer (Hach Lange, Wrocław, Poland). Distilled water was used as a control sample, and 500 µg/mL diclofenac was used as a positive control. As part of the analyses, three independent experiments were performed, in which each sample was tested in triplicate. Inhibition of protein denaturation by the analysed extracts was calculated using the equation:

% denaturation inhibition = (1 − As)/Ac × 100%

where:

As—the absorbance of the test sample,
Ac—the absorbance of the control sample.

#### 3.7.2. Assessment of Inhibition of Lipoxygenase Activity

Another test assessing the anti-inflammatory properties of the analysed extracts was the test assessing possibility of inhibiting activity of lipoxygenase enzyme. For this purpose, the methodology described by Ziemlewska et al. was applied [46]. In the first stage of analysis, 10 µL of test extracts (at concentrations 100–1000 µg/mL), 160 µL of PBS (100 mM), and 20 µL of soybean lipoxygenase solution (167 U/mL) were added to each well of a 96-well plate. The samples on the plate were mixed thoroughly on a rotary shaker and incubated at 25 °C for 15 min. Then, 10 µL of linoleic sodium was added to each well to initiate enzymatic reaction. Subsequently, the absorbance of the samples was measured every minute for a period of 5 min at λ = 234 nm using a microplate reader (Thermo Fisher Scientific, Waltham, MA, USA). Diclofenac at a concentration of 500 µg/mL was used as a standard inhibitor of lipoxygenase activity. Three independent experiments were performed, in which all concentrations of *B. officinalis* extracts were tested in triplicate. Percentage of inhibition of lipoxygenase activity was calculated from the equation:

% inhibition of lipoxygenase activity = (Ac − As)/Ac × 100%

where:

As—the absorbance of the test sample,
Ac—the absorbance of the control sample.

#### 3.7.3. Assessment of Inhibition of Proteinase Activity

The anti-inflammatory properties of the analysed extracts were also assessed by analysing the possibility of inhibiting another enzyme-proteinase. For this purpose, the methodology described by Juvekar et al. [47] and Gunathilake et al. [21] with minor modifications was used. Initially, a 1% trypsin solution was dissolved in Tris-HCl buffer (20 mM, pH = 7.4) and then mixed with the extract samples at concentrations of 100–1000 µg/mL. These mixtures were then incubated in an incubator at 37 °C for 5 min. In the next step, 0.8% (*w*/*v*) casein was added to the prepared samples and incubated for another 20 min. After this time, 70% perchloric acid was added to the samples to stop the enzymatic reaction. Diclofenac at a concentration of 500 µg/mL was used as a standard inhibitor. The samples were then centrifuged and absorbance at λ = 210 nm was measured using a DR600 UV–vis cuvette spectrophotometer (Hach Lange, Wrocław, Poland). A reaction buffer was used as a blank and a phosphate buffer solution was used as a control. As part of the analyses, three independent experiments were performed, in which all samples were tested in triplicate. The percent inhibition of proteinase activity was calculated using the equation:

% inhibition of proteinase activity = 100 × (1 − A_2_)/A_1_

where:

A_1_—the absorbance of the control sample,
A_2_—the absorbance of the test sample.

### 3.8. Anti-Ageing Activity

The anti-ageing properties of *B. officinalis* were assessed by determining the ability of the tested extracts to inhibit collagenase and elastase activity.

#### 3.8.1. Determination of Anti-Collagenase Activity

The study also included an assessment of the possibility of inhibiting the activity of collagenase—one of the matrix metalloproteinases involved in degradation of collagen. For this purpose, a fluorometric set was used (Abcam, ab211108, Cambridge, UK) and the determination was performed according to the manufacturer’s instructions and according to the procedure described earlier by Nizioł-Łukaszewska et al. [48]. The analyses were performed for all types of extracts in the concentration range from 100 to 1000 μg/mL. Measurements were conducted on a black 96-well plate with a clear flat bottom. In the first step of the assay, the collagenase (COL) was dissolved in the buffer provided with the kit (CAB). Then, test samples in appropriate concentrations were added to the COL and CAB. An inhibitor control sample was prepared by mixing a collagenase inhibitor (1,10-phenanthroline (80 mM)) with COL and CAB, and a control was prepared by mixing COL with CAB. CAB buffer was used as background control. The samples prepared in this way were incubated in the dark at room temperature for 15 min. A reaction mixture was then prepared by mixing the collagenase substrate with the CAB. Then, the prepared reaction mixture was added to all wells with analysed samples and thoroughly mixed on an orbital shaker. In the last step, fluorescence was measured at excitation wavelength 490 nm and emission 520 nm. Collagenase activity was measured in kinetic mode for 60 min at 37 °C. Three independent experiments were carried out in which all analysed samples were tested in duplicate. The ability to inhibit the activity of COL by the analysed extracts was calculated using the equation:

% relative COL inhibition = (enzyme control − sample)/enzyme control × 100

#### 3.8.2. Determination of Anti-Elastase Activity

Evaluation of the anti-ageing properties of *B. officinalis* extracts was also carried out with use of a fluorometric kit (Abcam, ab118971, Cambridge, MA, USA), which enables inhibition of the activity of enzyme-neutrophilic elastase (NE) to be assessed. The determinations were made in accordance with the manufacturer’s recommendations and the procedure previously described by Zagórska-Dziok et al. [49]. Fluorometric measurements were performed in a 96-well plate with black walls and a clear bottom. The analysis was performed for all types of extracts in the concentration range of 100–1000 µg/mL. Initially, solutions of the NE enzyme, NE substrate, and the inhibitor (succinyl-alanyl-alanyl-prolyl-valinochloromethylketone, SPCK) were prepared. The NE solution was then added to all wells on the microplate. In the next step, extracts at appropriate concentrations, the inhibitor, or the enzyme control (assay buffer) were added to separate wells. The prepared plates were placed in the dark and incubated at 37 °C for 5 min. Meanwhile, a reaction mixture was prepared by mixing assay buffer and NE substrate. The mixture prepared in this way was added to each well and the fluorescence was immediately measured at excitation wavelength λ = 400 nm and emission λ = 505 nm using a microplate reader (FilterMax F5, Thermo Fisher Scientific, Waltham, MA, USA). Fluorometric measurements of the tested samples were performed in kinetic mode (for 30 min at 37 °C). Three independent experiments were carried out in which all analysed samples were tested in duplicate. The effect of the individual concentrations of the tested extracts on NE activity was calculated using the equation:

% Relative Activity = (∆RFU inhibitor assay)/(∆RFU enzyme control) × 100%

### 3.9. Statistical Analysis

Values for the parameters assessed in this study were expressed as mean ± standard deviation (SD). Quantitative results of HPLC-DAD analysis were compared by one-way analysis of variance (ANOVA). The Tukey HSD (honestly significant difference) post hoc test was used to compare groups. The STATISTICA version 12 PL software package (StatSoft) was used for the HPLC-DAD analysis. The results of the in vitro analyses were compared by two-way analysis of variance (ANOVA), and the statistical analysis was performed using GraphPad Prism 8.0.1 (GraphPad Software, Inc., San Diego, CA, USA). A *p*-value < 0.05 was considered statistically significant.

## 4. Conclusions

Plant extracts containing polyphenolic compounds with a wide spectrum of biological activity have long been known and applied in medicine and in the pharmaceutical and cosmetics industries. Due to growing interest in skin care using topical products based on materials of natural origin, researchers are searching for plant sources rich in active substances, including flavonoids and phenolic acids. Attention to the high quality and safety of topical preparations for skin care and treatment of various skin conditions, as well as the trend to replace synthetic materials with natural ones, suggest the need for analyses of the phytochemical composition and effects of plant extracts.

The present study highlights the differences in phenolic content and biological activity of various *B. officinalis* extracts. The results indicate that borage extracts can be a valuable source of biologically active substances that reduce levels of free radicals, exert anti-inflammatory effects, and inhibit skin ageing processes. Owing to their lack of negative effect on the metabolic activity and viability in skin cells, borage extracts can be regarded as a safe material for use in topical preparations. Further research is needed to determine the precise mechanisms of action of these extracts, but the present research has shown that *B. officinalis* herb extracts may be a useful and important material with a broad spectrum of activity, particularly in the cosmetic and pharmaceutical industries.

## Figures and Tables

**Table 1:** Content (mg/100 g DW ± SD) of flavonoids in methanol and water–methanol (70:30) extracts of *B. officinalis* herb.

Flavonoids	Air-Dried Herb		Hot-Air-Dried Herb	
	Methanol Extract	Water:Methanol Extract (70:30)	Methanol Extract	Water:Methanol Extract (70:30)
Astragalin	147.40 ± 13.77 ^bcd^	48.96 ± 1.95 ^ac^	248.60 ± 12.47 ^abd^	61.54 ± 1.21 ^ac^
Kaempferol 4-glucoside	70.73 ± 11.58 ^bcd^	39.79 ± 1.38 ^acd^	141.08 ± 1.43 ^abd^	26.69 ± 1.36 ^ac^
Rutoside	77.09 ± 11.31 ^cd^	66.06 ± 2.72 ^cd^	95.71 ± 1.70 ^abd^	42.22 ± 0.65 ^abc^
Vitexin	40.84 ± 4.28 ^bd^	3.28 ± 0.30 ^ac^	42.84 ± 6.07 ^bd^	1.41 ± 0.08 ^ac^
Total content	336.06 ± 40.94 ^bcd^	158.09 ± 6.35 ^ac^	528.23 ± 21.67 ^abd^	131.86 ± 3.30 ^ac^

Values are the mean of three replicate determinations (*n* = 3) ± SD. ^a^ *p* < 0.05 vs. air-dried herb methanol extract; ^b^ *p* < 0.05 vs. air-dried herb water:methanol extract (70:30); ^c^ *p* < 0.05 vs. hot-air-dried herb methanol extract; ^d^ *p* < 0.05 vs. hot-air-dried herb water:methanol extract (70:30).

**Table 2:** Content (mg/100 g DW ± SD) of phenolic acids in methanol and water–methanol (70:30) extracts of *B. officinalis* herb.

Phenolic Acids	Air-Dried Herb		Hot-Air-Dried Herb	
	Methanol Extract	Water:Methanol Extract (70:30)	Methanol Extract	Water:Methanol Extract (70:30)
Caffeic acid	18.56 ± 0.94 ^bd^	28.66 ± 5.17 ^ac^	20.42 ± 5.29 ^bd^	25.13 ± 0.66 ^ac^
Chlorogenic acid	13.71 ± 4.78 ^bd^	33.90 ± 3.63 ^abc^	11.13 ± 2.76 ^bd^	23.63 ± 2.26 ^abc^
3,4-Dihydroxyphenylacetic acid	101.48 ± 5.48 ^bcd^	260.17 ± 8.68 ^ad^	243.61 ± 18.25 ^ad^	189.88 ± 6.79 ^abc^
Ferulic acid	171.50 ± 16.49 ^bcd^	9.95 ± 0.63 ^ac^	205.30 ± 6.64 ^abd^	7.95 ± 0.41 ^ac^
*p*-Hydroxybenzoic acid	4.68 ± 0.56 ^bcd^	6.64 ± 0.40 ^acd^	5.71 ± 0.59 ^abd^	5.85 ± 0.17 ^ab^
Protocatechuic acid	8.80 ± 1.23 ^c^	10.30 ± 0.23 ^cd^	4.16 ± 0.42 ^abd^	8.06 ± 0.25 ^bc^
Rosmarinic acid	1173.80 ± 14.03 ^bcd^	309.22 ± 14.34 ^acd^	1783.55 ± 54.07 ^abd^	227.58 ± 9.93 ^abc^
Syringic acid	17.81 ± 1.08 ^bcd^	10.72 ± 1.06 ^ad^	7.31 ± 1.19 ^ad^	0.96 ± 0.07 ^abc^
Total content	1510.34 ± 44.59 ^bcd^	669.56 ± 34.14 ^acd^	2281.19 ± 89.21 ^abd^	489.04 ± 20.54 ^abc^

Values are the mean of three replicate determinations (*n* = 3) ± SD. ^a^ *p* < 0.05 vs. air-dried herb methanol extract; ^b^ *p* < 0.05 vs. air-dried herb water:methanol extract (70:30); ^c^ *p* < 0.05 vs. hot-air-dried herb methanol extract; ^d^ *p* < 0.05 vs. hot-air-dried herb water:methanol extract (70:30).

## Data Availability

Not applicable.

## Sample Availability

Samples of the compounds are not available from the authors.
